# Spatial distribution and determinants of asymptomatic malaria risk among children under 5 years in 24 districts in Burkina Faso

**DOI:** 10.1186/s12936-018-2606-9

**Published:** 2018-12-07

**Authors:** Mady Ouédraogo, Sékou Samadoulougou, Toussaint Rouamba, Hervé Hien, John E. M. Sawadogo, Halidou Tinto, Victor A. Alegana, Niko Speybroeck, Fati Kirakoya-Samadoulougou

**Affiliations:** 1 Centre de Recherche en Epidémiologie, Biostatistiques et Recherche Clinique, Ecole de Santé Publique, Université libre de Bruxelles, Brussels, Belgium; 2 Institut de Recherche Santé et Sociétés, Faculté de Santé Publique, Université catholique de Louvain, Brussels, Belgium; 3 Pôle Epidémiologie et Biostatistique, Institut de Recherche Expérimentale et Clinique, Faculté de Santé Publique, Université catholique de Louvain, Brussels, Belgium; 4 Unité de Recherche Clinique de Nanoro, Institut de Recherche en Sciences de la Santé, Centre National de la Recherche Scientifique et Technologique, Ouagadougou, Burkina Faso; 5 Département de Santé Publique, Centre Muraz, Bobo-Dioulasso, Burkina Faso; 6 Geography and Environment, University of Southampton, Southampton, UK; 7 Flowminder Foundation, Stockholm, Sweden

**Keywords:** Burkina Faso, Bayesian, Malaria, Map, Health district, Spatial

## Abstract

**Background:**

In malaria endemic countries, asymptomatic cases constitute an important reservoir of infections sustaining transmission. Estimating the burden of the asymptomatic population and identifying areas with elevated risk is important for malaria control in Burkina Faso. This study analysed the spatial distribution of asymptomatic malaria infection among children under 5 in 24 health districts in Burkina Faso and identified the determinants of this distribution.

**Methods:**

The data used in this study were collected in a baseline survey on “evaluation of the impact of pay for performance on the quality of care” conducted in 24 health districts in Burkina Faso, between October 2013 and March 2014. This survey involved 7844 households and 1387 community health workers. A Bayesian hierarchical logistic model that included spatial dependence and covariates was implemented to identify the determinants of asymptomatic malaria infection. The posterior probability distribution of a parameter from the model was summarized using odds ratio (OR) and 95% credible interval (95% CI).

**Results:**

The overall prevalence of asymptomatic malaria infection in children under 5 years of age was estimated at 38.2%. However, significant variation was observed between districts ranging from 11.1% in the district of Barsalgho to 77.8% in the district of Gaoua. Older children (48–59 vs < 6 months: OR: 6.79 [5.62, 8.22]), children from very poor households (Richest vs poorest: OR: 0.85 [0.74–0.96]), households located more than 5 km from a health facility (< 5 km vs  ≥ 5 km: OR: 1.14 [1.04–1.25]), in localities with inadequate number of nurses (< 3 vs  ≥ 3: 0.72 [0.62, 0.82], from rural areas (OR: 1.67 [1.39–2.01]) and those surveyed in high transmission period of asymptomatic malaria (OR: 1.27 [1.10–1.46]) were most at risk for asymptomatic malaria infection. In addition, the spatial analysis identified the following nine districts that reported significantly higher risks: Batié, Boromo, Dano, Diébougou, Gaoua, Ouahigouya, Ouargaye, Sapouy and Toma. The district of Zabré reported the lowest risk.

**Conclusion:**

The analysis of spatial distribution of infectious reservoir allowed the identification of risk areas as well as the identification of individual and contextual factors. Such national spatial analysis should help to prioritize areas for increased malaria control activities.

**Electronic supplementary material:**

The online version of this article (10.1186/s12936-018-2606-9) contains supplementary material, which is available to authorized users.

## Background

Malaria remains a major public health problem in sub-Saharan Africa (SSA). The recent global malaria report estimated the annual number of malaria deaths as 445,000 [1] of which 90% occur in SSA [1]. Burkina Faso ranks 4th among Economic Community of West African States (ECOWAS) countries with high infant and child mortality rates [2]. However, between 1998 and 2015, the infant-juvenile mortality rate dropped from 219 to 82‰ [3]. In 2016, according to the statistics of the Ministry of Health of Burkina Faso, malaria was the main reason for medical consultation (45%) and accounted for 60% of inpatient and out-patient case burden at hospitals [4]. Malaria accounts for 46% of deaths in Burkina Faso hospitals [4]. Children under 5 years of age and pregnant women are at the highest risk.

The fight against malaria is a high priority of Burkinabe policymakers with the objective of reduction of 40% (about a twice) of malaria incidence by 2020 compared to 2015 and reduction of case-fatality rate of malaria among children under 5 years of age to less than 1% over the same period [5]. To achieve this goal, the National Malaria Control Programme (NMCP) of Burkina Faso advocates for vector control, effective case management and seasonal chemo-prevention in line with neighbouring countries in west Africa [6]. These activities are coordinated at the level of the health district, which are the operational entities in Burkina Faso’s health system.

Despite these efforts, the NMCP lacks national-level operational tools or maps that can guide malaria control in identifying high-risk districts. The previous global and continental level maps of malaria from 1997, 2014 and 2016 [7–9] are not specifically tailored the health district-level. Moreover, in a context where malaria is endemic, historical maps do not allow policy makers to target high-risk areas for intervention needs. Indeed, the estimation of risk in these maps is presented as gridded layers based on interpolation. While visually appealing in terms of presenting sub-national variation, they cannot be easily summarized by programme managers or national-planning decision-makers [10]. Local authorities are, therefore, deprived of effective tools for prioritizing interventions, and financial resources [11]. In addition to the scarcity of malaria risk mapping, there is only little knowledge of the determinants of malaria at the community level in the context of Burkina Faso [12]. While studies conducted in some countries SSA tend to show that the involvement of CHW in the health system contributes to reduce the risk of malaria transmission [13, 14] in Burkina Faso, little is known about the issue. The objective of this study was to produce maps at the 24 health districts that can guide malaria control programme in Burkina Faso and to examine the determinants of malaria asymptomatic infection in children under five.

## Methods

### Study design and area

That is a cross sectional study conducted in the 24 health districts of the six regions of Burkina Faso selected as part of the baseline survey on “Evaluation of the Impact of Results-Based Financing in Burkina Faso”. The population of these 24 districts is estimated at about 6,015,562 and ranges from 141,830 for Nanoro District to 285,922 for Gaoua District [15]. The rainfall varies across the country (from Southwest to Northern region) with 1045 mm and 611 mm of annual rainfall record in Gaoua and Gourcy health district respectively. Furthermore, the malaria transmission intensity varies across the country and influenced by climatic factors, malaria transmission is holoendemic (Gaoua), hyperendemic (Boromo, Toma, Sapouy, Batié Dano and Diébougou) and mesoendemic (the of 24 districts) [16]. *Plasmodium falciparum* is responsible for majority of diagnosed malaria cases. The number of people living below the poverty line is high in these 6 regions. Indeed, the poverty rate ranges from 36.1% (East Center) to 70.4% (North) [17].

### Sampling procedures and data collection

This study used data from the baseline survey “evaluation of the impact of pay for performance on the quality of care in Burkina Faso”. It was carried out in partnership with several institutions namely, the Institute of Public Health of the University Heidelberg, the University Hospital of Montreal, Centre Muraz, Bobo Dioulasso, World Bank Office located in Ouagadougou, HRITF (Trust Fund for Innovation in Health Outcomes), and the Programme of health development support (PADS) of the Ministry of Health of Burkina Faso. The report of the survey was published on the World Bank website [18].

The cross-sectional survey was carried out in six regions (Boucle de Mouhoun, Centre-East, Centre-North, Centre-West, North and South-West) of the country between October 2013 and March 2014, which included 24 health districts and 560 health facilities. The survey consisted of two components: a household survey and a health facility survey among CHWs. The choice of regions was made with reasoning (sampling) and was guided by the low-level rate of maternal and child health indicators in these six regions. Four districts per region were randomly selected.

To implement the household survey, a two-stage stratified cluster sampling was performed. For each health facility, the first stage of the sampling entailed randomly selecting a village and creating a sample frame that included all households which had at least one pregnant woman or a woman who delivered in the last 2 years. Within each frame, the second stage entailed a systematic sampling of 15 households. In total 7844 households were surveyed. For each household, data on socio-demographic and economic factors as well as health status of individuals, health care-seeking behaviours and associated expenditures (adults and children) were collected. These data were collected using a structured electronic questionnaire form designed on a personal digital assistant (PDA).

The questionnaire was in French and has been administered in French. However, if the respondent does not understand French and the interviewer does not understand the interviewee’s mother language, a translator was asked. The interviewers were trained during 2 weeks on the objectives of the survey and on the electronic data collection procedures. For this purpose, an interviewer’s manual for data collection (with PDA) has been edited. This training of the interviewers was co-organized and carried out by the Centre Muraz and the University of Heidelberg teams. For data collection, the interviewers were accompanied by their direct supervisors. A second level of supervision was provided by the data managers (controllers). A third level of investigator control was provided by a team of supervisors consisting of the MURAZ Center and the Heidelberg team.

For malaria cases, a rapid diagnostic test (RDT) using Sd Bioline (65, Borahagal-ro, Giheung-gu, Yongin-si, Gyeonggi-do, 446–930, Republic of Korea) was performed on all children under 5 years of age (N = 10,245) in the households surveyed.

For the survey among CHWs, a random sample of 3 CHWs was performed in each health facility. When a health facility was covered by fewer than 4 CHWs, all CHWs were interviewed. A total of 1387 CHWs were interviewed using a face to face questionnaire and included principal themes concerning roles and responsibilities, satisfaction, motivation, and compensation including delays in payment of wages. A structured paper-based questionnaire was used and then entered using double data entry at Centre MURAZ.

### Variables of interest

#### Response variable

The response variable in the study was asymptomatic malaria infection in children under 5 years of age detected with a rapid diagnostic test (RDT) during the survey. Since the prevalence of malaria transmission can be assessed through serological markers [19], the RDT Sd Bioline which detect histidine-rich protein II (HRP-II) was used. Serological methods used for malaria diagnosis are RDTs, which can detect HRP-II and/or parasitic lactate dehydrogenase (pLDH). The antibodies against malaria antigens are sensitive biomarkers of malaria exposure at the population level and can be used to identify areas of high risk of malaria transmission, to assess transmission levels, to monitor variations throughout time or the impact of interventions, and to confirm malaria elimination [20].

#### Independent variables

The independent variables used in this study are factors that may influence asymptomatic malaria infection in children as frequently reported in previous malaria studies in SSA countries [7, 8, 10, 21–26]. The socio-demographic covariates were: sex (male/female), age (0–59 months), use of insecticide-treated nets (ITN) the day before the child’s survey (yes/no), mother’s education level (no-educated/educated), number of insecticide-treated nets for 2 persons in the household, distance to closest health facility (less than 5 km/more than 5 km), household’s standard of living (very poor, poor, moderate rich, rich, very rich), place of residence (urban/rural), district and region of residence.

At the community level, the key explanatory variables were: number of health workers by health facility (less than 3 nurses/more than 3 nurses), number of children received by CHWs for health care and sensitization provided by CHWs to households (yes/no). These later variables are modifiable characteristics upon which action can be taken.

The meteorological variables used were the monthly average temperature per district and the monthly average precipitation per district according to the months corresponding to the period of the study. To take into account the transmission season of malaria, the time was divided into two seasons: a high season from October to December and a low transmission season from January to March [27]. These variables are widely used in malaria mapping to describe seasonal variation [7, 8]. Temperature and precipitation data were obtained from Global Climate Data [28].

### Statistical analyses

Two approaches were used to analyse these data: (1) descriptive analysis was used to describe the study population and to assess the association between asymptomatic malaria infection and categorical variables we performed a Chi square test; (2) spatial analysis was used to describe the spatial heterogeneity of the malaria distribution in 24 districts in Burkina Faso and to identify health districts with high risk of malaria.

### Spatial modelling methods

The Moran test (Moran I Index) was used to analyse the spatial autocorrelation of asymptomatic malaria infection in children in the 24 health districts of Burkina Faso. Moran’s I statistic tests the null hypothesis that the prevalence of asymptomatic malaria infection observed in a district (i) is independent of those observed in neighbouring districts (j). The neighbourhood was defined by associating each district with its immediate neighbour (Queen contiguity criterion). A modelling method by taking into account the existence of significant spatial autocorrelation between asymptomatic malaria in order to avoid biasing the estimation of the confidence intervals of the parameters was used [29–32].

Briefly, a hierarchical Bayesian spatial logistic model was used to predict whether a child *i* living in district *j* is infected by malaria parasite [33–36]. The general form of this model was:

1 $$y_{ij} \sim {\text{Bernoulli}}\left( {\pi_{ij} } \right)$$

2 $$\log \left( {\pi_{ij} } \right) = \left( {X\beta } \right)_{ij} + \xi_{j}$$

3 $$\log \left[ {\frac{{\pi_{ij} }}{{1 - \pi_{ij} }}} \right] = \beta_{0} + \mathop \sum \limits_{p = 1}^{P} \beta_{p} x_{pij} + u_{j} + v_{j}$$

In this general linear model, the logit (*π*_*ij*_) is used to model the probability of success as a linear combination of observed individual characteristics (*x*_*ij*_) and contextual (*x*_*j*_) characteristics associated with an unobserved specific effect of the district *ξ*_*j*_. *ξ*_*j*_ can be assumed as random intercepts indicating how much the risk to have asymptomatic malaria infection in each district varies compared to the mean risk for the entire 24 districts (*β*_0_) after taking into account observed effects for all covariates. This district-specific effect can be decomposed into a sum of a spatial random effect (structured *u*_*j*_) and a nonspatial random effect (unstructured *v*_*j*_).

This modelling was implemented in a Bayesian framework. The posterior probability distributions of marginal effect of the parameters were obtained using the integrated nested Laplace approximation (INLA), which is a valid and effective alternative to Markov chain Monte Carlo (MCMC) methods [37–39]. To facilitate and simplify the utilization of the study findings by policy makers, the probability of excess risk, the baseline probability of asymptomatic malaria infection, as well as the fraction of the variance attributed to spatial autocorrelation were computed.

Furthermore, the probability of excess risk in the district was used to generated maps by categorizing the risk according to Richardson recommendation [40]. Richardson's classification consists of classifying the study areas into three categories: a district has a high risk of asymptomatic malaria if the posterior probability distributions of contracting the disease is greater than 0.8, low if the probability is less than 0.2, and medium if the probability is between 0.2 and 0.8

The deviance information criterion (DIC) [41] was used to assess the performance of the full model versus the null model. The posterior probability distribution of a parameter from the model was summarized using odds ratio (OR) and 95% credible interval (95% CI). The details of the modelling are presented in the Additional file 1.

## Results

### Descriptive analysis

About 38.2% of children had an asymptomatic malaria infection. Table 1 shows the distribution of children under 5 according to socio-demographic characteristics, and environmental variables. Overall 52.1% of the children were female. The distribution of the study population by age shows that 28.4% were between 12 and 24 months and only 12.4% were between 24 and 36 months. Thus, the distribution of the sample was homogeneous in all age groups except for the 12–24 months age group.

**Table 1:** Sociodemographic characteristics of the respondents

Modality	N	Percentage
Sex		
Male	4905	47.9
Female	5340	52.1
Age of child in months		
0–5	1543	15.1
6–11	1569	15.3
12–23	2909	28.4
24–35	1266	12.4
36–47	1549	15.1
48–59	1409	13.7
Place of residence		
Urban	745	7.3
Rural	9500	92.7
Region		
Boucle du Mouhoun	1983	19.4
Centre-East	1053	10.3
Centre-North	2069	20.2
Centre-West	2176	21.2
North	2225	21.7
South-West	739	7.2
Sleeping under an insecticide-treated net		
No	1744	17.3
Yes	8350	82.7
Household socioeconomic status		
Very poor	1872	18.3
Poor	1866	18.2
Moderate poor	2005	19.6
Rich	2211	21.6
Very rich	2291	22.3
Education level of the mother		
No education	9875	96.5
Educated	360	3.5
Number of insecticide-treated nets by household		
Less than 1 for 2	9251	90.3
Equal or more than 1 for 2	994	9.7
Distance from health facility		
Less than 5 km	6057	59.2
Equal or more than 5 km	4168	40.8
Number of health workers per health facility		
Less than 3 nurses	1391	13.6
Equal/more than 3 nurses	8854	86.4
Number of children for health care by CHW		
None	4216	41.2
1–10	4316	42.1
≥ 11	1713	16.7
Provide Health Education Services (CHW)		
Yes	6500	63.8
No	3680	36.2
Temperature in °C		
Less than 27.5	8281	80.8
Equal or more than 27.5	1964	19.2
Rainfall in mm		
< 50	4780	48.4
50–100	4616	46.7
≥ 100	487	4.9
Season		
January–March 2014	4701	45.9
October–December 2013	5544	54.1

The majority of households (90.3%) surveyed had less than one ITN for 2 persons and 82.7% of children in all age groups examined had slept under an ITN the night before the survey. The distribution of the sample according to household characteristics showed that 22.4% of the children surveyed lived in richer households and 18.3% in the most disadvantaged (very poor) households. In terms of accessibility to health facilities, 40.8% of the children surveyed lived in households located more than 5 km from the closest health facility. 96.5% of the mothers of the children surveyed had no formal education. The sample distribution according to community variables indicates that 58.8% of children lived in localities where CHWs have given health care to sick children and 36.2% in localities where CHWs performed household visits for health sensitization.

Table 2 shows differences for asymptomatic malaria infection among children by region and district of residence. Asymptomatic malaria infection was most prevalent in children in the South West region (63.7%) and least prevalent in children in the Centre North region (24.6%).

**Table 2:** Prevalence of asymptomatic malaria by region and health district

	Asymptomatic infection N (%)
Total	3916 (38.2)
Boucle du Mouhoun region	793 (40.0)
Boromo	131 (58.0)
Nouna	271 (31.6)
Solenzo	269 (40.7)
Toma	122 (51.5)
Centre-East region	381 (36.2)
Manga	52 (32.9)
Ouargaye	196 (45.9)
Tenkodogo	124 (30.4)
Zabré	9 (15.0)
Centre-North region	509 (24.6)
Barsalgho	10 (11.1)
Kaya	241 (24.4)
Kongoussi	186 (27.5)
Ziniaré	72 (23.0)
Centre-West region	950 (43.7)
Koudougou	523 (39.2)
Nanoro	26 (21.3)
Réo	114 (38.1)
Sapouy	287 (68.2)
North region	812 (36.5)
Boussé	28 (13.8)
Gourcy	162 (29.9)
Ouahigouya	557 (46.0)
Yako	65 (24.1)
South-West region	471 (63.7)
Batié	132 (68.4)
Dano	52 (61.2)
Diébougou	210 (58.0)
Gaoua	77 (77.8)

In addition, the highest proportion of asymptomatic malaria infection was observed in Gaoua district (77.8%) and lowest in Barsalogho district (11.1%). Among the 24 districts, the prevalence of asymptomatic malaria infection was higher than the mean of 24 districts (38.2%) in the following 11 districts: Gaoua, Batié, Boromo, Toma, Solenzo, Ouargaye, Koudougou, Sapouy, Ouahigouya, Dano and Diébougou.

Table 3 presents the factors associated with asymptomatic malaria infection in 24 districts in Burkina Faso. The crude model showed that the child’s age, household socioeconomic status  and distance are significantly associated with asymptomatic malaria infection. Thus, children in the age group of 36–47 months were more at risk (OR: 7.45, 95% CI [6.19, 8.98]) than younger age groups (6 months). Regarding household socioeconomic status, it appears that children from very rich households were less likely to be affected by asymptomatic malaria infection (OR: 0.88, 95% CI [0.77, 0.90]) compared to their counterparts in very poor households. The results also indicate that children in households more than 5 km from a health facility were more likely to have asymptomatic malaria infection (OR: 1.22, 95% CI [1.11, 1.33]) compared to those located within 5 km of the health facility.

**Table 3:** Factors associated with asymptomatic malaria infection in 24 health districts in Burkina Faso

	Crude odd ratio (95% credible interval)	Adjusted odd ratio (95% credible interval)
Sex		
Male	1	1
Female	0.97 [0.89,1.06]	0.97 [0.88,1.05]
Age of child in months		
0–5	1	1
6–11	2.81 [2.33,3.41]	2.82 [2.33,3.42]
12–23	5.06 [4.26,6.01]	5.07 [4.28,6.04]
24–35	7.14 [5.89,8.65]	7.12 [5.87,8.65]
36–47	7.45 [6.19,8.98]	7.52 [6.11,8.88]
48–59	6.76 [5.60,8.17]	6.79 [5.62,8.22]
Place of residence		
Urban	1	1
Rural	1.67 [1.40,1.98]	1.67 [1.39,2.01]
Total insecticide-treated net for 2 persons in the household		
Less than 1 for 2	1	1
Equal or more than 1 for 2	1.04 [0.95.1.14]	0.94 [0.81,1.09]
Sleeping under an insecticide-treated net		
No	1	1
Yes	0.99 [0.88.1.11]	1.01 [0.89,1.13]
Household standard of living		
Very poor	1	1
Poor	0.96 [0.84,1.10]	0.92 [0.79,1.06]
Moderate poor	0.97 [0.85,1.11]	0.92 [0.79,1.06]
Rich	0.95 [0.83,1.08]	0.91 [0.79,1.05]
Very rich	0.88 [0.77,0.90]	0.85 [0.74,0.96]
Education level of the mother		
No education	1	1
Educated	0.92 [0.73,1.16]	0.81 [0.62,1.06]
Distance to health facility		
< 5 km	1	1
≥ 5 km	1.22 [1.11,1.33]	1.14 [1.04,1.25]
Number of health workers per health facility		
< 3	1	1
≥ 3	0.69 [0.61,0.79]	0.72 [0.62,0.82]
Number of children for health care by CHW		
None	1	1
1–10	0.99 [0.89,1.10]	1.00 [0.89,1.12]
≥ 11	0.99 [0.86,1.13]	0.98 [0.85,1.13]
Provide Health Education Services (CHW)		
Yes	1	1
No	1.12 [1.02,1.23]	1.07 [0.97,1.19]
Temperature in °C		
< 27.5	1	1
≥ 27.5	1.37 [0.72,2.65]	1.15 [0.73,1.82]
Precipitation in mm		
< 50	1	1
50–100	1.02 [0.40,2.55]	1.55 [1.04,2.30]
≥ 100	1.59 [0.60,4.21]	2.15 [0.87,5.27]
Season		
January–March 2014 (low transmission of malaria)	1	1
October–December (2013) (high transmission of malaria)	1.21 [1.06,1.38]	1.27 [1.10,1.46]

The results also indicate the presence of a significant association between the number of nurses in health facilities, health sensitization service provided by CHWs to households and the asymptomatic malaria infection in children. Localities with fewer than three nurses at the health facility had the highest risk of infection as compared to other localities (OR: 0.69, 95% CI [0.61, 0.79]). In addition, children from households without health sensitization were more likely (OR: 1.12, 95% CI [1.02, 1.23]) to have asymptomatic malaria infection than those who benefited from sensitization. Children from rural areas had a higher risk of asymptomatic malaria infection (OR: 1.67, 95% CI [1.40, 1.98]) compared to those from urban areas. Results also indicate that between October and December (high transmission season of malaria) children were more at risk (ORL 1.21, 95% CI [1.06, 1.38]) than between January and March (low transmission season of malaria).

The results of the multivariable analysis confirm the bivariate analysis findings and indicate that the age of the child, the standard of living of the household, the distance from the child’s household to the nearest health facility, the number of nurses, place of residence and season were significantly associated with asymptomatic malaria infection. Therefore, children older than 6 months were at higher risk to have asymptomatic malaria infection than their younger counterparts (48–59 months vs < 6 months OR: 6.79 95% CI [5.62, 8.22]). The same is true for children living in households located more than 5 km from a health facility (OR 1.14, 95% CI [1.04, 1.25]) compared to those whose households are within 5 km. In addition, the richest children were less exposed to asymptomatic malaria than the poorest (OR: 0.85, 95% CI [0.74, 0.96]). Compared to children living in localities where health facilities meet the World Health Organization standard for number of health professionals, those in other localities had a higher risk of asymptomatic malaria infection (OR 0.72, 95% CI [0.62, 0.82]). Children from rural areas (OR: 1.67, 95% CI [1.39, 2.01]) were also more at risk than those in urban areas. Similarly, children surveyed between October and December were more at risk than those surveyed between January and March (OR: 1.27 [1.10, 1.46]).

Table 4 presents the Moran’s I Index and the indicators to assess the performance of the full model versus the null model. Asymptomatic malaria in children under 5 years of age were spatially correlated (Moran index = 0.046 with p value < 0.001). Table 4 also indicates that the full model is better matched to describe the spatial distribution of asymptomatic malaria in 24 districts in Burkina than the null model. Indeed, the improvement of full model in terms of deviance ($\bar{D}$) offsets its complexity (pD), thus leading to a smaller deviance information criterion (DIC) of 840.9 points than the null model. In addition, covariates seem to have little explanatory influence because their inclusion in the model led to a 3% reduction in inter-district variability $\left( {\sigma_{u}^{2} + \sigma_{v}^{2} } \right)$ of malaria morbidity among children. This inter-district variability is largely attributable to the structured spatial effect (99.4%).

**Table 4:** Summary of model fit

Statistic	Null model	Full model
Moran I index	0.046	p < 0.001
Posterior mean of the deviance ($\bar{D}$)	12,842.07	11,977.19
Effective number of parameters (pD)	22.02	46.00
Deviance information criterion (DIC)	12,864.10	12,023.19
Between district variability of malaria $\sigma_{u}^{2} + \sigma_{v}^{2}$	933.99	906.33
Proportion of variance attributed to spatial autocorrelation (ɸ)	0.995	0.994

**Fig. 1:**
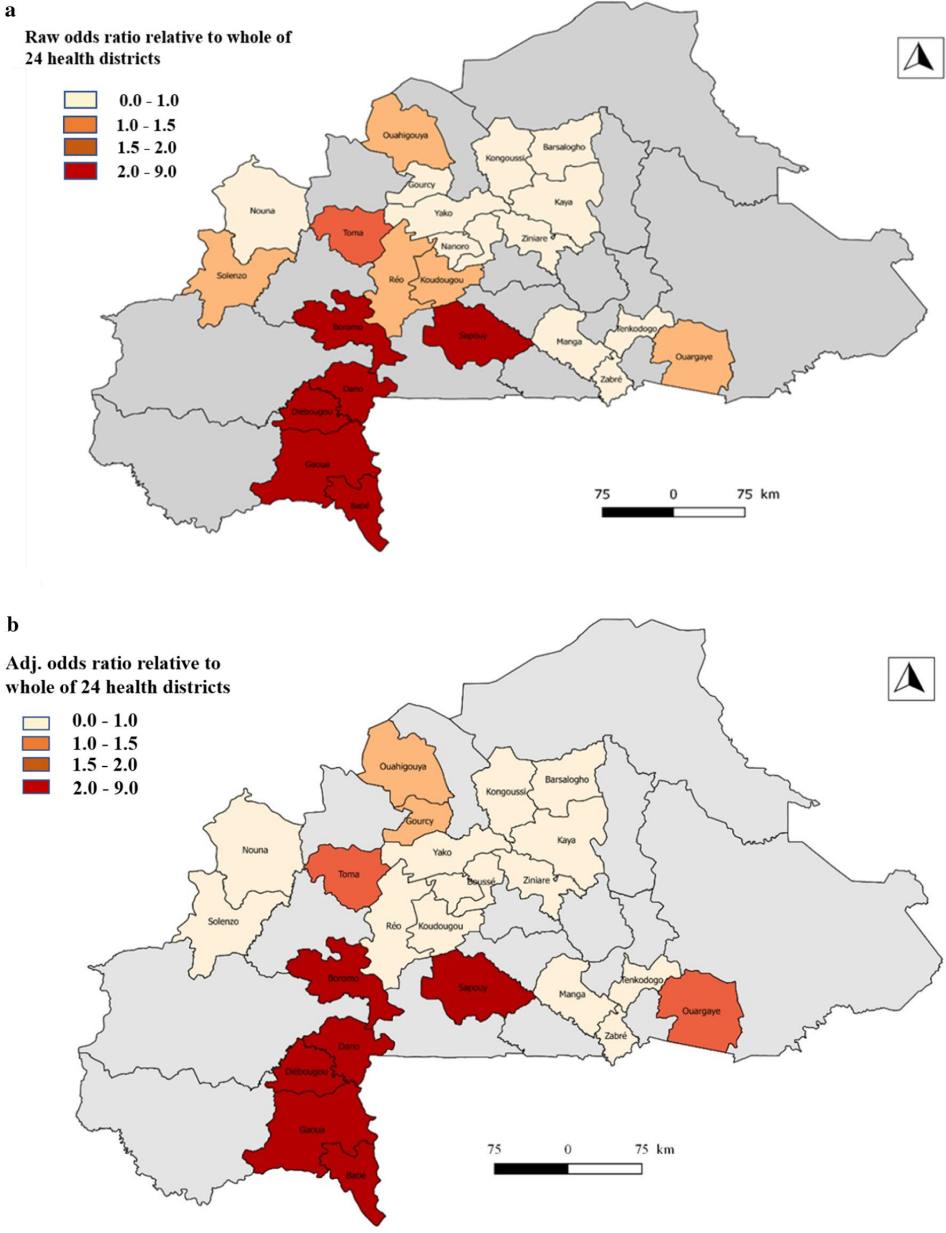
Odds Ratio comparing the estimated risk of asymptomatic malaria in a district to the mean of 24 districts asymptomatic malaria risk. Map A (null model). Map B (adjusted model for socio demographic characteristic, environmental and climatic variables)

**Fig. 2:**
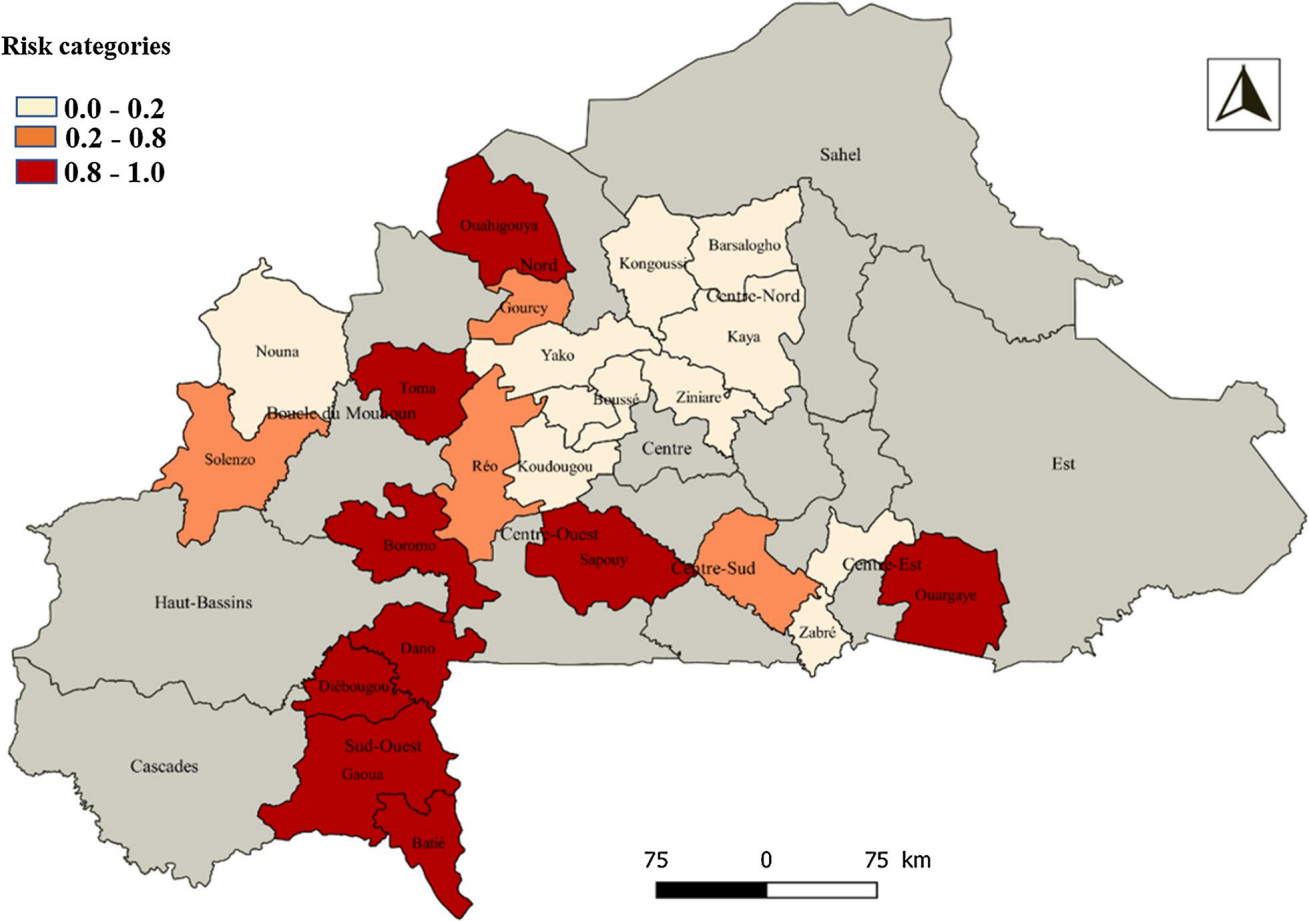
Risk categories based on Richardson’s classification. Pr(*ξ*_*j*_ >  0/*y*) > 0.8, 0.2 < Pr(*ξ*_*j*_ > 0/*y*) ≤ 0.8, 0.0 ≤ Pr(*ξ*_*j*_ > 0/*y*) ≤ 0.2

Figure 1 shows the spatial distribution of asymptomatic malaria in 24 health districts in Burkina Faso. Map A (null model) presents the odds ratio of malaria morbidity among children in the 24 districts and Map B (adjusted model) shows the adjusted odds ratio of malaria morbidity in the districts after taking into account the effect of the predictor variables. Map (B) allows for identifying the districts with a higher risk of malaria compared to the national risk. From the analysis of the two maps, it appears that the configuration of the spatial distribution of malaria has varied slightly. Categorization of risk based on Richardson’s classification [40] identified nine districts that reported significantly higher risks. These are the districts of Batié, Boromo, Dano, Diébougou, Gaoua, Ouahigouya, Ouargaye, Sapouy and Toma (Fig. 2 and Table 5).

**Table 5:** Higher and Lower Risk Districts

Excess of risk [0.8–1.0]^a^	Medium risk [0.2–0.8]^b^	Low risk [0.0–0.2]^c^
Batié	Gourcy	Barsalogo
Boromo	Manga	Boussé
Dano	Réo	Kaya
Diébougou	Solenzo	Kongoussi
Gaoua		Koudougou
Ouahigouya		Nanoro
Ouargaye		Nouna
Sapouy		Tenkodogo
Toma		Yako
		Zabré
		Ziniaré

^a^Pr(*ξ*_*j*_ > 0/*y*) > 0.8

^b^0.2 < *Pr*(*ξ*_*j*_ > 0/*y*) ≤ 0.8

^c^0.0 ≤ Pr(*ξ*_*j*_ > 0/*y*) ≤ 0.2

**Fig. 3:**
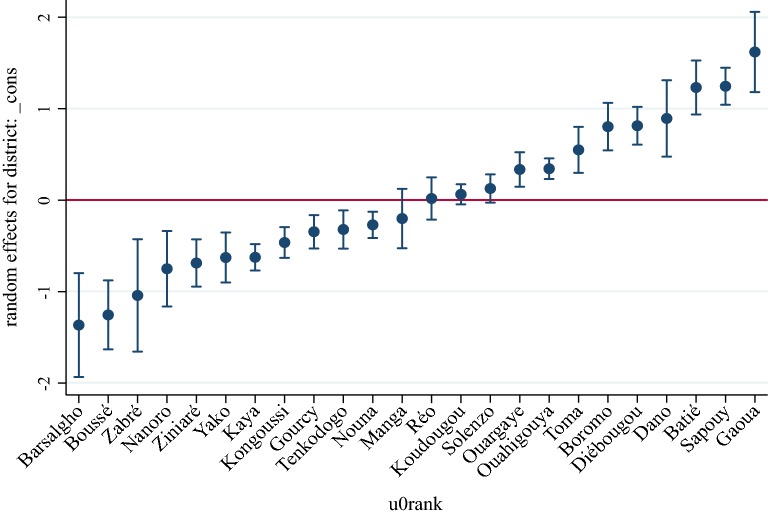
District specific risk

Figure 3 ranks district-specific risk estimates (ξ_j_) on a logit scale with a 95% credible interval.

## Discussion

The findings suggest that the probability of asymptomatic malaria varies from 0.93 to 0.99 in high-risk districts (e.g. Sapouy and Gaoua) and less than 0.07 in the low-risk districts (e.g. Yako and Koudougou). According to the analysis, a child from Burkina Faso had a 14-fold chance of infection in high-risk districts compared to in low-risk districts. Children in high-risk districts are infected throughout the year or were  asymptomatic individuals/carriers. These asymptomatic malaria cases are important reservoirs of malaria, responsible for year-round transmission [42, 43]. The reported parasitic carrying rates reported in the study area were high compared to neighbouring countries [44] and confirmed the high endemicity of malaria in the study area.

Asymptomatic malaria risk cartography identified nine districts in which the risks were significantly higher than the mean risk of 24 health districts, confirming those of the bivariate analysis. Four of them were located in the South-west (Batié, Dano, Diebougou, Gaoua), two in the Boucle du Mouhoun region (Boromo, Toma), one in the North (Ouahigouya), one in the Central-east (Ouargaye) and one in the Central-west (Sapouy). This risk classification can be considered as robust because it takes into account socio-demographic/economic, meteorological and community factors. The heterogeneity of the spatial distribution of asymptomatic malaria in the population (reservoir) reported in this study could be relevant information for control programmes for the reduction of global and local malaria transmission.

Indeed, in the context of limited resources, and in order to achieve the goal of reducing mortality rates in children under 5 by 2020 [6], the government of Burkina Faso could plan using the risk estimates and probability maps presented in this study as tools to prioritize control. Using the results of the study for planning will contribute to the reduction of under-five mortality and enable the country to achieve SDG target 3.2 which is to reduce the mortality rate of children under 5 years of age to 25 per 1000 live births by 2030. This risk mapping activity is of great importance for policy makers, as in many countries with limited resources, the success of malaria control often depends on careful planning of resources and delivery of interventions in remote areas [45].

Furthermore, asymptomatic malaria is also associated with type of residence, with higher prevalence in rural areas. This could be explained by the vector life cycle, biology and impact of urbanization on transmission (for urban environments). Indeed, the literature indicates that rural and remote areas provide not only conducive environmental and climatic conditions for the complete *Plasmodium* life cycle [46, 47], but also an appropriate site for the reproduction of the *Anopheles* vectors [48]. The variation of prevalence with age suggests that immunity plays a key role in the variations of asymptomatic malaria risk among young children in line with other studies [49].

The following factors were important determinants of infection rates. Firstly, children in poorer households depicted higher infection rates. This observation highlighted that poverty influenced malaria prevalence by creating conditions (poor housing, lack of knowledge, negative health behaviours) that favours the spread of infectious diseases and limiting access to prevention and treatment. Secondly, distance to nearest health centre as well as low staffing (nurses) was associated with higher infection. Children from households located more than 5 km far from a health centre were more likely to contract asymptomatic malaria, confirming findings reported by other authors [50]. This high risk of asymptomatic malaria could be explained by the low use of health services by households due to geographical inaccessibility of health centres [51]. Studies conducted in Malawi [52] and Tanzania [53] confirm the findings. The workload of health workers is often cited as a major constraint that impedes sessions of behaviour change information. Thirdly, there was no significant difference in asymptomatic malaria between children using bed nets and those not use it. Previous studies suggest infection in asymptomatic individuals could occur outside home, or, in the evening before sleeping under a bed net [26, 49, 54–56].

There are some limitations to the study. The sample of the study was representative of the 24 health districts not of all the districts in Burkina Faso. Moreover, the results could be influenced by the study design of the main survey which lead to an over-representation of children under the age of 2. Indeed, immunity protection attenuates the age-prevalence curve. However, the present analysis gives an estimation of reference level of the result-based financing indicators before the project implementation. In addition, the results presented in this article are largely based on the prevalence of parasitaemia using RDT, which may overestimate the number of false negatives [57]. The study, however, is among the first studies that provide a probabilistic characterization of asymptomatic malaria spatial distribution at health district-level in Burkina Faso. Another important limitation to consider was the constitution of the sampling frame for household selection that could lead to selection bias. In fact, it included all households with at least one pregnant woman or one woman who gave birth in the last 2 years.

Despite these limitations, this study contributes to the literature. Indeed, it represents the first study to provide a probabilistic characterization of the spatial distribution of malaria in the districts of Burkina Faso. The Bayesian Hierarchical Modeling Framework used in this article was useful as it has resulted in robust estimates, and decision-makers may consider using these results during planning and monitoring programme.

## Conclusion

This study is among the few studies taking into account both community and spatial distributions of asymptomatic malaria infection in 24 districts Burkina Faso. The analysis showed that the prevalence of asymptomatic malaria is influenced by the geography but also climatic and sociodemographic/economic characteristics. That means that its eradication will require not only biological interventions (proximal) but also social interventions (distal). This study also demonstrates the utility of the Bayesian hierarchical model for understanding the distribution of asymptomatic malaria. This disease mapping technique could be used systematically in the planning and evaluation of malaria elimination efforts.

## Additional file

**Additional file 1.**Statistical modeling details

## Abbreviations

CHW: Community-based Health Worker
CI: Credibility Intervals
CMA: "Centre Médical avec Antenne chirurgicale" Medical Centre with Surgical Antenna
HF: Health Facility
DIC: Deviance Information Criterion
RBF: Results Based Financing
GLM: Bayesian Generalized Linear Model
INLA: Integrated Nested Laplace approximation
MRF: Markov Random Field Models
PDA: Personal Digital Assistant
RDT: Rapid Diagnostic Test
