# Supplementary material for: Spatial distribution and determinants of asymptomatic malaria risk among children under 5 years in 24 districts in Burkina Faso
Source: Malar J. 2018 Dec 7;17:460. doi: 10.1186/s12936-018-2606-9 (PMC6286519; doi:10.1186/s12936-018-2606-9)
Supplement: Supplementary file 1 — Additional file 1.Statistical modeling details [file 12936_2018_2606_MOESM1_ESM.docx]

**Additional file**

**Additional file 1:** Statistical modeling details

*Modeling method*

To predict asymptomatic malaria infection in children living in district j, a "Bayesian generalized linear model" (GLM) with random effects was used [1-4]. This model assumes that a child with asymptomatic malaria infection results from the realization of Bernoulli test with the probability of success (positive malaria) $\pi_{ij}$ (equation1). In the binomial spatial regression model (equations 2 and 3), The $logit(\pi_{ij}$) is used to model the probability of success as a linear combination of observed individual characteristics ($x_{ij}$) and contextual ($x_{j}$) characteristics plus an unobserved district specific effect$\xi_{j}$. The $\xi_{j}$ can be understood as random intercepts indicating how much the risk to have a asymptomatic malaria infection in each district varies compared to the national average risk ($\beta_{0}$) after taking into account the observed effects of all covariates ($\sum_{p=1}^{P} \beta_{p}x_{pij}$). This district-specific effect can be decomposed into a sum of a spatial random effect (structured $u_{j}$) and a nonspatial random effect (unstructured $v_{j}$). The combination $u_{j}+v_{j}$ allows to take into account the spatial dependence in the modeling process [5].

$y_{ij}\sim Bernoulli\left( \pi_{ij} \right)$ (1)

$\log\left( \pi_{ij} \right)={(X\beta)}_{ij}+\xi_{j}$ (2)

$\log\left[ \frac{\pi_{ij}}{1-\pi_{ij}} \right]=\beta_{0}+\sum_{p=1}^{P} \beta_{p}x_{pij}+u_{j}+v_{j}$ (3)

$\beta\sim Normal \left( 0,{10}^{-4} \right)$ (4)

$\frac{u_{j}}{u_{k}}, j\neq k, \tau_{\mu}\sim\mathrm{Normal}\left( \frac{1}{\mathcal{N}_{j}}\sum_{j\sim k} u_{k}, \frac{1}{\mathcal{N}_{j}\tau_{\mu}} \right)$ (5)

$v_{j}\sim\mathrm{Normal}\left( 0, \tau_{v} \right)$ (6)

${log(\tau}_{u})\sim log\mathrm{Gamma}\left( 1, {10}^{-3} \right)$ (7)

$log(\tau_{v})\sim log\mathrm{Gamma}\left( 1, {10}^{-3} \right)$ (8)

The prior probability distributions for unknown random parameters are specified as follows. The vector of the regression parameters β, corresponding to the q modalities of the qualitative variables for the child i of the district j, is normally distributed with 0 as mean and weak precision (equation 4).

The spatial structured random effect $u_{j}$follows a conditional autoregressive distribution (CAR) whose mean and precision depend on the structure as well as the size $\mathcal{N}_{j}$of the first order neighbours (j ~ k) of each district (Eq 5).

The non-spatial random effect (ustructured) $v_{j}$ follows a normal distribution with a changeable structure (equation 6). The accuracies of $u_{j}$ et $v_{j}$ (Eqs 7 et 8) follow a logGamma distribution (1, 0.001) [6].

The neighbourhood matrix is constructed using Queen's contiguity criteria [7].

This spatial modeling approach using Markov chains (MRF) was used in recent years in Malawi [8, 9] to analyse data on malaria and in Kenya [10] to analyse data on prenatal care. The authors report good performance of the MRF model compared to other spatial models in statistics or econometrics [11].

The posterior distributions of the marginal effect of the model parameters are obtained using the integrated nested Laplace approximation (INLA), which is a valid and efficient alternative to the Markov chain Monte Carlo (MCMC) simulation methods [12-14]. To facilitate and simplify the utilization of the study findings by policy makers, values such as the probability of excess risk, the baseline probability of asymptomatic malaria infection, as well as the fraction of the variance attributed to spatial autocorrelation were computed.

The Deviance Information Criterion (DIC) [15] was used to evaluate the performance of the full model versus the empty model.

The posterior distribution of a model parameter was summarized using its mean and 95% credible interval.

**References**

1. Kruschke JK: What to believe: Bayesian methods for data analysis. Trends in cognitive sciences 2010, 14:293-300. https://doi.org/10.1016/j.tics.2010.05.001.

2. Kruschke JK: Bayesian data analysis. Wiley Interdisciplinary Reviews: Cognitive Science 2010, 1:658-676. <https://doi.org/10.1016/j.tics.2010.05.001>.

3. Vaughn BK: Data analysis using regression and multilevel/hierarchical models, by Gelman, A., & Hill, J. Journal of Educational Measurement 2008, 45:94-97. http://lac-essex.wdfiles.com/local--files/meetings1213/gelman_1.pdf. Accessed 2

4. Gelman A, Hill J: Data analysis using regression and multilevelhierarchical models. Cambridge University Press New York, NY, USA; 2007.

5. Lawson AB: Bayesian disease mapping: hierarchical modeling in spatial epidemiology. CRC press; 2013.

6. Lunn D, Jackson C, Best N, Thomas A, Spiegelhalter D: The BUGS book: a practical introduction to Bayesian analysis. CRC Press; Chapman & Hall, 2012, pp. 339.

7. Bivand RS, Pebesma EJ, Gomez-Rubio V, Pebesma EJ: Applied spatial data analysis with R. Springer; 2008.

8. Chirombo J, Lowe R, Kazembe L: Using Structured Additive Regression Models to Estimate Risk Factors of Malaria: Analysis of 2010 Malawi Malaria Indicator Survey Data. PLoS One 2014, 9: e101116.

https://doi.org/10.1371/journal.pone.0101116.

9. Stevenson JC, Pinchoff J, Muleba M, Lupiya J, Chilusu H, Mwelwa I, Mbewe D, Simubali L, Jones CM, Chaponda M, et al: Spatio-temporal heterogeneity of malaria vectors in northern Zambia: implications for vector control. Parasit Vectors 2016, 9. <https://doi.org/10.1186/s13071-016-1786-9>.

10. O’Meara WP, Platt A, Naanyu V, Cole D, Ndege S: Spatial autocorrelation in uptake of antenatal care and relationship to individual, household and village-level factors: results from a community-based survey of pregnant women in six districts in western Kenya. Int J Health Geogr. 2013;12:55. <https://doi.org/10.1186/1476-072X-12-55>.

11. Chitunhu S, Musenge E: Spatial and socio-economic effects on malaria morbidity in children under 5years in Malawi in 2012. Spat Spatio-temporal Epidemiol 2016, 16:21-33. <https://doi.org/10.1016/j.sste.2015.11.001>

12. Bivand RS, Gómez-Rubio V, Rue H: Spatial data analysis with R-INLA with Some Extensions. 2015. J Stat Software. 2015;63:3387.

https://brage.bibsys.no/xmlui/bitstream/handle/11250/276910/Bivand_Gomez-Rubio_Rue.pdf?sequence=3. Accessed 05 octobre 2017.

13. Blangiardo M, Cameletti M: Spatial and spatio-temporal Bayesian models with R-INLA. John Wiley & Sons; 2015.

14. Martino S, Rue H: Case Studies in Bayesian Computation using INLA. In Complex Data Modelling and Computationally Intensive Statistical Methods. Mantovan O, Secchi P, Eds. Springer, Milan, 2010.

15. Spiegelhalter DJ, Best NG, Carlin BP, Van Der Linde A: Bayesian measures of model complexity and fit. Journal of the Royal Statistical Society: Series B (Statistical Methodology) 2002, 64:583-639.
